# Modest association between health literacy and risk for peripheral vascular disease in patients with type 2 diabetes

**DOI:** 10.3389/fpubh.2022.946889

**Published:** 2022-08-24

**Authors:** Shang-Jyh Chiou, Yen-Jung Chang, Kuomeng Liao, Chih-Dao Chen

**Affiliations:** ^1^Department of Health Care Management, National Taipei University of Nursing and Health Sciences, Taipei City, Taiwan; ^2^Department of Health Promotion and Health Education, National Taiwan Normal University, Taipei City, Taiwan; ^3^Department of Endocrinology and Metabolism, Zhongxiao Branch, Taipei City Hospital, Taipei City, Taiwan; ^4^Department of Family Medicine, Far Eastern Memorial Hospital, New Taipei City, Taiwan

**Keywords:** health literacy, peripheral vascular disease, type 2 diabetes, diabetes management, survey

## Abstract

**Objective:**

Health literacy plays a crucial role in managing chronic health conditions. Previous studies have revealed the positive relationship between health literacy and diabetes knowledge but few studies have focused on peripheral vascular disease (PVD) in diabetes in relation to health literacy in diabetes management. This study investigated the relationship between the risk for PVD and health literacy level with other determining factors among patients with type 2 diabetes.

**Method:**

We conducted a survey on health literacy using the Mandarin Multidimensional Health Literacy Questionnaire in the department of metabolism and endocrine systems at a regional hospital in northern Taiwan from December 2021 to May 2022 and obtained data from the hospital's health information system (HIS) from 2013 to 2020 to identify occurrences of PVD (*n* = 429). We performed logistic regression analysis to identify the relationship between PVD events and health literacy levels (overall and in five separate subdimensions) adjusted with other variables.

**Results:**

A longer duration of diabetes increased the risk for PVD events (*P* = 0.044 and 0.028). In terms of health literacy, the overall level was not significant; however, the dimension of higher levels of health literacy in acquiring health information increased the risk for PVD events (*P* = 0.034). Other variables were not significantly associated with the risk for PVD events.

**Conclusion:**

This study examined the risk for PVD events in terms of the duration of diabetes and provided evidence across the range of dimensions of health literacy concerning the ability to control diabetes. Those with a higher level of health literacy may be more aware of their disease situation, seek and cooperate with their healthcare providers earlier, and have more opportunities to be made aware of their health status from regular checkups than those with inadequate health literacy. These results may help providers make available more self-management tools that are adequate and sustainable for diabetes patients with poor health literacy.

## Introduction

Type 2 diabetes mellitus (T2DM) is a serious public health issue prevalent in aging societies, especially when the condition is not well-controlled. It increases the risk for micro- and macro-complications (such as retinopathy, dialysis, and peripheral vascular disease [PVD]) (1). The first step in diabetes management involves lifestyle changes in diet and physical activities (e.g., taking regular exercise) together with self-monitoring of blood glucose (SMBG) and medication adherence. These efforts in diabetes management involve long-term work and require patients to cooperate with medical teams or with their healthcare providers to obtain the optimal glycemic level. Studies show that patients with T2DM can enjoy a good quality of life and a lower risk for future complications if they follow diabetes management guidelines well (2, 3).

Chronic disease management studies have found that health literacy, defined as the capability to acquire, understand, and apply health knowledge, plays a crucial role in managing chronic health conditions (4, 5). Better outcomes are identified in patients with higher health literacy levels (6, 7). Furthermore, the performance of basic reading and numerical tasks requires them to function in the healthcare environment, which helps patients take the wheel in their disease condition. Low (or inadequate) health literacy may lead to a gap in communication with the medical team, an information gap in the use of medical devices for SMBG, or a functional gap in diabetes management in daily life. These gaps could lead to poor diabetes management (e.g., worse glycemic control), create a risk for diabetes-related problems (such as hypoglycemia), or contribute to worse outcomes, especially in terms of chronic diseases. In addition, studies have identified a connection between low health literacy and poor knowledge in terms of diabetes (8), which is also associated with poor self-related health (9) and more utilization of healthcare (10, 11).

The risk for PVD in diabetic patients is occurring earlier at an increasing rate and is becoming more severe and diffuse (12). The high blood sugar levels commonly associated with diabetes can, over time, damage the blood vessels. Poor diabetes management leads to increased morbidity and mortality in PVD due to dysfunction, inflammation, and hypercoagulability in the vascular system. One study in the UK found that 1.2% of newly diagnosed patients with T2DM also had PVD (13), suggesting that intensive targeting of glycemic control is needed. Few studies have focused on the prevalence of PVD in diabetes concerning issues of health literacy in diabetes management and the consequences of PVD, which leads patients to control their diabetes better.

We hypothesize that, in patients with type 2 diabetes, having better health literacy may enhance their diabetes care management and reduce the risk of complications (such as PVD) in the future. Therefore, this study investigated the relationship between the risk for PVD at different health literacy levels among patients with T2DM. The results may be useful for medical teams caring for patients with T2DM who otherwise have poor health literacy to conceive useful strategies in diabetes care.

## Methods

### Population

This was a cross-sectional survey conducted in a department of metabolism and endocrine systems at a northern regional hospital in Taiwan from December 2021 to May 2022. Before the survey, all patients provided written informed consent, which included the use of medical records from the hospital's health information system (HIS) for the analysis. All participating patients had T2DM and answered the questionnaire voluntarily; those with mental disorders or cognitive impairments, who were unable to provide informed consent or participate in the survey, were excluded. After completing the survey, we compensated the participants with NTD 100 gift cards. In all, after data correction (missing or double ID), 429 patients from the interview by trained staff were used in the analysis. The Institutional Review Board of the hospital approved the study design, which conformed to the ethical principles of the 2008 Declaration of Helsinki.

### Variables

#### The outcome variable

The outcome we used in this study was the record of pulse volume recording for each patient from 2013 to 2020, as applicable, drawn from the hospital's HIS to identify events of PVD. Because participants had multiple medical records over the study period, we defined PVD events as those occurring when participants had PVD records in the abnormal figure throughout the study period. The abnormal range of PVD was defined as ankle brachial index <0.8.

#### Independent variable

We not only used using the Mandarin Multidimensional Health Literacy Questionnaire (MMHLQ) for the measurement of health literacy, but also collected participant demographic, and diabetes-related information. The demographic variables included age, sex, education, and marital status. The diabetes-related information included SMBG (Do you regularly monitor your blood glucose when not in hospital? Yes/No), diabetes control methods (medication, insulin, diet [following the suggestions of the medical team], in terms of regular exercise [three times a week for at least 30 min per session], smoking behavior [Yes/No], and drinking behavior [Yes/No]), diabetes duration (<1 year, 1–5 years, 6–10 years, or >11 years), any family history of diabetes (Yes/No), ability to takeregular exercise, ability to follow a diet plan, ability to follow treatments, ability to perform SMBG, and responses to the Diabetes Knowledge Questionnaire (DKQ).

To assess their ability to follow regular exercise and diet plans, we asked the participants to evaluate the degree of comprehension in understanding the diabetes control plans (in diet and exercise) from the medical teams on a scale of 0–10. To assess the ability to follow treatment and perform SMBG, we asked participants to respond to the following items: understanding the treatment plan (Yes/No), knowing how to take the medicine (Yes/No), understanding the drug information (Yes/No), knowing how to use the glucometer (Yes/No), identifying the device information from the glucometer (Yes/No), and knowing the level of fasting plasma glucose (Right/Wrong). The DKQ is a 24-item questionnaire that records the understanding of the cause of diabetes with complications, blood glucose levels, diet, and physical activity, has three response options “yes,” “no,” and “don't know” and has been reported to have optimal reliability and validity (14). The higher the score, the better the knowledge of the disease (The highest achievable score was 24). In addition, the Cronbach's alpha reliability of DKQ-24 was 0.796 (alpha if item deleted was 0.784–0.797) which represented an acceptable range (15).

To test health literacy, we used the MMHLQ, which includes 20 items in 5 dimensions (acquiring, understanding, assessing, applying, and communication). Every item has four scales (very difficult 1, difficult 2, easy 3, and very easy 4), and a transforming formula was used to obtain scores ranging from 0 to 50 in the five dimensions and the total. Later, we cataloged these scores into four levels: inadequate (≤25), limited/problematic (25 ≤ 33), sufficient (33 ≤ 42), and excellent (42 ≤ 50). Several studies have demonstrated that the MMHLQ is an adequate tool for health literacy (16–18). In addition, the Cronbach's alpha reliability of MMHLQ was 0.79 (alpha if item deleted was 0.77–0.94) which represented an acceptable range.

### Statistics

To better understand the distribution of health literacy, we provided the original response to every item in the five dimensions across the four-level distribution. We were also interested in the factors associated with health literacy, and we used the chi-square test and ANOVA to test for significant differences. Finally, we performed logistic regression to explore the relationship between PVD events and health literacy levels (both overall and across the five sub-dimensions) adjusted for other variables. Note that we only considered a high correlation between education and health literacy; thus, we only included health literacy in the logistic regression model. All statistical data were analyzed using SAS 9.3.1 (SAS Institute, Cary, NC) and SPSS 20.0 (SPSS Inc., Chicago, IL). The significance level was set to *P* < 0.05.

## Results

The basic characteristics of the participants are presented in Table 1. The majority were male (59.7%), married (71.3%), had no smoking or drinking behavior, had a family history of diabetes (70.6%), and had a diabetes duration of >11 years (40.7%). Most used medication, and over half followed a controlled diet and took regular exercise, although only 46% of them performed SMBG. The abilities in regular exercise, diet plan, treatment, and SMBG were 7.67, 7.98, 3.86, and 3.31, respectively. The mean DKQ score was 15.4.

**Table 1:** The basic characteristics from the survey (*n* = 429).

**Variables**		***N***	**%**
Sex	Male	256	59.7
	Female	167	38.9
Education	Primary	60	14.5
	Junior high	48	11.2
	Senior high	175	40.9
	College	109	25.5
	graduate	34	7.9
Marriage	Single	58	16.8
	Married	246	71.3
	Others	41	11.9
SMBG	No	231	54.0
	Yes	197	46.0
Control methods	Medication	404	94.2
	Insulin	121	28.2
	Diet	254	59.2
	Regular exercise	222	51.7
Smoking behavior	Yes	91	21.2
Drinking behavior	Yes	51	11.9
Diabetes years	<1	33	7.7
	1–5	125	29.3
	6–10	95	22.2
	>11	174	40.7
Family history	Yes	303	70.6
**Age**		60.47 ± 12.58	(range 18–88)
Ability in exercise		7.67 ± 2.67	(10 is highest)
Ability in diet plan		7.98 ± 2.34	(10 is highest)
Ability in treatments		3.86 ± 0.50	(4 items)
Ability in SMBG		3.31 ± 0.96	(4 items)
DKQ		15.4 ± 4.28	(24 is highest)

SMBG, Self-Monitoring of Blood Glucose; DKQ, diabetes knowledge questionnaire.

Health literacy was determined as follows: over 25% of patients expressed difficulty in searching for the needed health information from the Internet (acquiring), 38% of patients expressed difficulty in judging the health information for trust from the Internet, 26% of patients expressed difficulty in determining the health information for consistency with others (assessing), 28% of patients expressed difficulty in applying the health information to choose the treatment (applying), and 26% of patients expressed difficulty in proposing the desired treatments to my providers (communication). However, over 95% of patients expressed easy or very easy understanding in the dimension of health literacy (Table 2 with red color). We used the scores to divide the patients into four levels: inadequate, limited/problematic, sufficient, and excellent. We found that combining the sufficient and excellent levels returned 69.9, 91.1, 54.3, 65.9, and 77.2% patient competence in acquiring, understanding, assessing, applying, and communication, respectively. Overall, 12.8, 32.4, 39.2, and 54.8% of patients had inadequate, limited/problematic, sufficient, and excellent health literacy skills, respectively (Table 3).

**Table 2:** The distribution of survey items and divided levels from health literacy in MMHLQ.

**Dimension**	**Variable**	**Very difficult**		**Difficult**		**Easy**		**Vary easy**	
		***n***	**%**	***n***	**%**	***n***	**%**	***n***	**%**
Acquiring health information	1 Seeking the knowledge related to disease	16	3.8	36	8.5	276	64.9	97	22.8
	2 Acquiring the information related to daily life	11	2.6	25	5.9	273	64.2	116	27.3
	3 Searching the needed health information from the internet	69	16.3	41	9.7	196	46.2	118	27.8
	4 Desire to know the report content from the health exam report	7	1.7	34	8.0	267	63.0	115	27.1
Understanding health information	5 Understanding the drug bag info	4	0.9	19	4.5	266	62.6	136	32.0
	6 Follow the medical team guideline to take care the disease			13	3.1	**273**	**64.4**	**138**	**32.5**
	7 Understand the explanation from the medical team			5	1.2	**260**	**61.2**	**160**	**37.6**
	8 Follow the package insert from the drug bag			19	4.5	**250**	**59.0**	**155**	**36.6**
Assessing health information	9 Determining the health information solving the health problems	13	3.1	83	19.5	273	64.2	56	13.2
	10 Determining the health information suiting for myself	21	4.9	66	15.5	286	67.3	52	12.2
	11 Determining the health information for the consistency with others	25	5.9	87	20.5	266	62.7	46	10.8
	12 Judging the health information for trusty from the internet	56	13.2	108	25.5	197	46.5	63	14.9
Applying health information	13 Applying the health information to understand the disease process	25	5.9	73	17.2	279	65.8	46	10.8
	14 Applying the health information to copy with the disease	25	5.9	61	14.4	288	68.1	49	11.6
	15 Applying the health information to understand the health exam outcomes	29	6.8	54	12.7	282	66.5	59	13.9
	16 Applying the health information to choice the treatments	27	6.4	91	21.6	255	60.4	49	11.6
communication	17 Proposing the desired treatments to my providers	**12**	**2.8**	**95**	**22.5**	241	57.0	75	17.7
	18 confirming the understanding of medical guideline with medical team	2	0.5	21	5.0	299	70.5	102	24.1
	19 Discussing the treatments with my providers	3	0.7	50	11.8	255	60.0	117	27.5
	20 Raising questions immediately to medical teams with any explanations from the medical team			21	5.0	247	58.3	156	36.8

MMHLQ, Mandarin Multidimensional Health Literacy Questionnaire. Bold values are higher percentages.

**Table 3:** The scores of five dimensions with overall and the distribution of health literacy levels in MMHLQ.

**Dimension**	**Mean ±SD**	**inadequate**		**Limited/** **problematic**		**Sufficient (S)**		**Excellent (E)**		**S+E**
		***n***	**%**	***n***	**%**	***N***	**%**	***n***	**%**	**%**
Acquiring	34.26 ± 10.0	90	21.0	39	9.1	212	49.4	88	20.5	69.9
Understanding	38.06 ± 8.6	19	4.4	19	4.4	264	61.5	127	29.6	91.1
Assessing	29.77 ± 10.3	150	35.0	46	10.7	203	47.3	30	7.0	54.3
Applying	30.39 ± 10.9	110	25.6	36	8.4	243	56.6	40	9.3	65.9
Communication	35.46 ± 8.1	54	12.6	44	10.3	238	55.5	93	21.7	77.2
Overall	33.52 ± 8.1	55	12.8	139	32.4	168	39.2	67	15.6	54.8

MMHLQ, Mandarin Multidimensional Health Literacy Questionnaire; SD, standard division.

We also identified the association between the other variables we used in the study and health literacy for the overall score and scores for the five subdimensions from chi square or ANOVA test (^*^significant level, *P* < 0.05). Younger individuals, those with higher education levels, and those with higher DKQ scores were found to have better health literacy levels (Table 4). Positive associations (+ in Table 4) were also observed between the performance of regular exercise, following the diet plan, and conducting SMBG and better health literacy levels, especially in acquiring, understanding, and assessing health information. However, we did not find a significant association between health levels and PVD risk (*P* = 0.355) (Table 5). In addition, we provided details in the supplemental file (see Supplementary Tables 1–6).

**Table 4:** The relationship between overall and sub-dimensions health literacy levels (MMHLQ) with other variables.

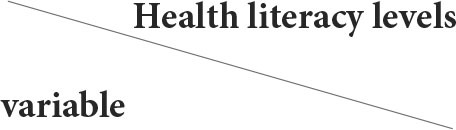	**Overall**	**Acquiring**	**Understanding**	**Assessing**	**Applying**	**Communication**
	***P***	***P***	***P***	***P***	***P***	***P***
**S**ex	0.078	0.038*	0.309	0.255	0.331	0.665
Age	<0.001*	<0.001*−	0.015*−	<0.001*−	<0.001*−	<0.001*−
Education	<0.001*	<0.001*+	0.002*+	<0.001*+	<0.001*+	<0.001*+
Marriage	0.005*	0.013*	0.02*	0.086	0.008*	0.032*
Doing SMBG	0.498	0.253	0.754	0.775	0.487	0.818
Diabetes years	0.034*	0.003*	0.352	0.001*	0.097	0.638
Family history	0.023*	0.110	0.053	0.063	0.025*+	0.384
Health problems	0.807	0.698	0.701	0.957	0.126	0.371
Ability in Regular Exercise	0.089	0.021*+	0.026*	0.018*+	0.719	0.004*
Ability in diet plan	<0.001*	<0.001*+	0.022*	0.007*+	0.302	0.185
Ability in treatments						
Understanding the plan	0.058	0.099	0.36	0.045*	0.001*+	0.048*
Know how to take it	0.205	0.137	0.812	0.427	0.529	0.619
Understanding the drug info	0.381	0.161	0.415	0.264	0.566	0.125
Ability in SMBG						
Knowing to use the devices	0.002*	<0.001*+	0.025*+	0.002*+	0.536	0.059
Knowing the device info	0.007*	0.002*+	0.026*+	0.041*+	0.262	0.055
Knowing the level	<0.001*	<0.001*+	0.023*+	<0.001*+	0.019*+	0.27
DKQ	<0.001*	<0.001*+	<0.001*+	<0.001*+	<0.001*+	<0.001*+

MMHLQ, Mandarin Multidimensional Health Literacy Questionnaire; SMBG, Self-Monitoring of Blood Glucose; DKQ, diabetes knowledge questionnaire; ANOVA test, Chi-square test.

* Significant level, P < 0.05.

**Table 5:** The distribution between overall health literacy levels (MMHLQ) with PVD.

		**Health literacy levels**				***P***
		**Inadequate**	**Limited**	**Sufficient**	**Excellent**	
PVD	Yes	34	98	118	42	0.355
	No	17	32	38	21	

PVD, Peripheral vascular disease.

Chi-square test (χ^2^ = 3.247 degree of freedom: 3).

We used two multivariable logistic regression models to explore the relationship between PVD events and health literacy levels with other variables. We tested the overall health literacy level and five subdimensions of health literacy level separately in the model, and only the dimension of acquiring health information was significant. Therefore, in Model 1, we used the overall health literacy level, and in Model 2, we used acquired health information because most participants had better scores in this health literacy sub-dimension. We found that a longer diabetes duration increased the risk for PVD events (*P* = 0.044 and 0.028) (Table 6). For health literacy, the overall level was not significant in Model 1; however, a higher level of acquiring health information increased the risk for PVD events in Model 2 (*P* = 0.034). Other variables were not significantly associated with the risk for PVD events.

**Table 6:** The relationship between PVD events with health literacy adjusted with other variables from logistics regression.

		**Model 1: Overall health literacy**				**Model 2: acquiring health information dimension**			
		**OR**	**95% LO**	**HU**	***P***	**OR**	**95% LO**	**HU**	***P***
Age		1.024	0.994	1.055	0.122	1.024	0.992	1.057	0.144
Sex	Male	0.521	0.268	1.010	0.053	0.506	0.258	0.994	0.048
Marriage	Single				0.203				0.364
	Married	0.462	0.184	1.161		0.545	0.216	1.379	
	Others	0.379	0.113	1.274		0.451	0.133	1.529	
Doing SMBG	Yes	0.614	0.342	1.104	0.103	0.589	0.328	1.059	0.077
Smoking	Yes	0.743	0.349	1.585	0.443	0.756	0.350	1.634	0.477
Drinking	Yes	1.013	0.454	2.263	0.975	1.101	0.486	2.496	0.818
Health literacy	Inadequate				0.298				0.034*
	Limited/problematic	0.551	0.224	1.354		1.363	0.469	3.957	
	Sufficient	0.662	0.264	1.661		0.553	0.233	1.315	
	Excellent	1.127	0.387	3.280		1.512	0.527	4.342	
Diabetes years	<1				0.044*				0.028*
	1–5	2.928	0.570	15.043		2.642	0.506	13.795	
	6–10	6.152	1.163	32.533		6.368	1.187	34.151	
	>11	5.964	1.203	29.559		5.762	1.158	28.670	
Family history	Yes	0.898	0.467	1.727	0.746	0.850	0.440	1.641	0.628
Ability in Exercise		1.054	0.931	1.193	0.407	1.064	0.938	1.206	0.335
Ability in diet		0.912	0.793	1.049	0.198	0.903	0.785	1.038	0.152
Ability in treatments		1.506	0.763	2.976	0.238	1.525	0.780	2.983	0.217
Ability in SMBG		0.953	0.653	1.390	0.802	0.935	0.638	1.372	0.732
DKQ		1.014	0.937	1.098	0.729	1.022	0.942	1.108	0.600

MMHLQ, Mandarin Multidimensional Health Literacy Questionnaire; SMBG, Self-Monitoring of Blood Glucose; DKQ, diabetes knowledge questionnaire; OR, odds ratio.

Logistic regression (chi-square: 37.267, df = 23, P = 0.03 in model 1, chi-square = 42.025, df = 23, P = 0.009 in model 2).

* Significant level, P < 0.05.

## Discussion

In this study, we found that the longer the patients have diabetes the more likely they are to experience a PVD even while the overall health literacy levels not significant. Only modest significance was found in the acquisition of health information for the health literacy dimension, and possible explanations did not have sufficient statistical power to identify any health literacy effects in this cross-sectional survey or imply that other factors or pathways need to be explored in further research. Interestingly, those who had a better record in acquiring health information health literacy levels had an increased risk for PVD events. It may be that those with better levels of acquiring health information had better health awareness in their diabetes management and had more opportunity to be made aware of their health status from regular checkups.

We also found that younger individuals had higher levels of health literacy and higher education levels. This result is consistent with many previous studies on its positive associations with age and education levels (19–23). However, mixed results for health literacy have also been found in other studies, relating to differences in measurement, tools, or specific diseases. Those who have better health literacy levels in acquiring health information (understanding health information) and assessing health information dimensions indicated a better ability to understand the merit of regular exercise and diet plans, as well as the ability to perform SMBG. One possible explanation is that patients with T2DM need to understand, assess, and implement health information related to diet, exercise, and SMBG to change their lifestyle, and poor health literacy impedes diabetes self-management to achieve optimal glycemic control (24, 25). However, other studies have indicated weak or no association with health literacy (26, 27). Interestingly, no significant association was found with health literacy and the ability to treat in medication. A possible explanation for this is that patients relied on the authority of providers and, most of them trusted the information that was provided most of the time. Patients with T2DM who had higher health literacy levels had better DKQ scores, which demonstrates the vital role that health literacy plays in diabetes knowledge (28). Clinical teams should understand the positive relationship between health literacy and diabetes knowledge and pay closer attention to detecting the patient's ability to understand and apply health information imparted by diabetes education materials. For this reason, educational materials should be provided or modified to adjust for different health literacy levels.

Our study confirms the increased risk for PVD events with longer diabetes duration, which is consistent with other studies (29, 30). Peripheral vascular disease can cause lower limb amputation or mortality, especially in people with T2DM, due to the risk factors for DM—hypertension, smoking, and hyperlipidemia—along with other traditional risks, such as age or low kidney function (31, 32). At-risk indicators include an older patient cohort, diabetes duration of longer than 10 years, high HbA1c, obesity, and neuropathy (33).

A modest significant effect was found in acquiring health information on health literacy levels associated with PVD events. Clinical staff should identify patients with inadequate or limited health literacy levels and provide easy-to-understand educational materials on diet and exercise plans to help them control their diabetes. In addition, we found positive associations between diet, exercise plan, and SMBG with diabetes knowledge and high levels of acquiring, understanding, and assessing health information. We should conceive useful strategies to help diabetic patients improve their capacity to apply health information and communication, such as mobile eHealth technology; to address health problems, especially among the older adult population, and to overcome these adoption barriers to suffering on the internet.

## Limitations

There are several limitations to address. First, although the MMHLQ is reliable and valid in clinical settings, we still need to compare the results with other measurements. However, the application of several health literacy measurements (such as the Test of Functional Health Literacy in Adults, Literacy Assessment for Diabetes, or Diabetes Numeracy Test) not only requires to be considered in terms of patient loading and under different construction conceptions but also faces the challenge of translation and employment in the generality or specialty in disease. However, the distribution of health literacy levels determined by the MMHLQ is acceptable. Second, the cross-sectional survey was limited to establishing a temporal association and diabetes care is long-term work, therefore, one survey can only reflect the information from the patient's diabetes management at that time. This information may not be sufficient to evaluate certain specific situations or the long-term aspects of the patient's diabetes care. Third, our results were obtained using data from a single center and may not be generalizable to other diabetic patients. In addition, patients in the hospital participated in the survey voluntarily and gave written consent; thus, we need to consider the possibility of selection and recall biases, which may have affected the results. These participants may have more health awareness of their disease situation; so they may have more positive factors in their diabetes management. Finally, considering the limited time in this study for answering the survey in the older adults group with type 2 diabetes, some items in the survey (for example, smoking, and drinking behavior) used the dichotomous scale without details which may underestimate the effect on outcomes. Further studies will be needed to provide evidence to clarify relationships in these items.

## Conclusions

More evidence was identified regarding the different dimensions of health literacy levels and the ability to control diabetes. A certain number of older adult patients with type 2 diabetes need assistance to search for, judge, determine, and apply health information acquired from the internet. Providing trustworthy and easy-to-understand health information from modern technology may be vital for diabetes care management. In addition, those who have a higher health literacy level may be more aware of their disease situation and seek and cooperate with their providers earlier than those who have inadequate health literacy. These results may help providers increase adequate and sustainable self-management tools in diabetes patients with poor health literacy.

## Data availability statement

The datasets presented in this study can be found in online repositories. The names of the repository/repositories and accession number(s) can be found below: Survey Research Data Archive (SRDA) in Academia Sinica in Taiwan.

## Ethics statement

The studies involving human participants were reviewed and approved by the Institutional Review Board of the Taipei City Hospital Research Ethics Committee. The patients/participants provided their written informed consent to participate in this study.

## Author contributions

S-JC and Y-JC contributed to the development of study methodology and study conceptualization. KL and C-DC provided clinical input. S-JC is the guarantor of this work and, as such, had full access to all the data in the study and takes responsibility for the integrity of the data and the accuracy of the data analysis. All authors contributed to interpretation of results, reviewed and drafting the manuscript and approved the final version.

## Funding

This work was supported by the Ministry of Science and Technology (MOST 109-2410-H-227-001-SSS). The funder had no role in study design, and analysis, or decision to publish.

## Conflict of interest

The authors declare that the research was conducted in the absence of any commercial or financial relationships that could be construed as a potential conflict of interest.

## Publisher's note

All claims expressed in this article are solely those of the authors and do not necessarily represent those of their affiliated organizations, or those of the publisher, the editors and the reviewers. Any product that may be evaluated in this article, or claim that may be made by its manufacturer, is not guaranteed or endorsed by the publisher.
