# Supplementary material for: Modest association between health literacy and risk for peripheral vascular disease in patients with type 2 diabetes
Source: Front Public Health. 2022 Aug 24;10:946889. doi: 10.3389/fpubh.2022.946889 (PMC9448987; doi:10.3389/fpubh.2022.946889)
Supplement: Supplementary file 1 [file Data_Sheet_1.docx]

Supplemental file

Table S1 the variables with significant associations with different overall health literacy levels

|  |  | inadequate | Limited/ problematic | sufficient | excellent | p |
| --- | --- | --- | --- | --- | --- | --- |
|  |  | N(%)  mean | N(%)  mean | N(%)  mean | N(%)  mean |  |
| Age |  | 69.44 | 63.72 | 56.86 | 55.40 | <0.001 |
| Education | Primary | 24(38.7) | 27(43.5) | 11(17.7) | 0(0) | <0.001 |
|  | J high | 7(14.6) | 21(43.8) | 14(29.2) | 6(12.5) |  |
|  | S high | 18(10.3) | 57(32.6) | 78(44.6) | 22(12.6) |  |
|  | College | 5(3.5) | 34(23.8) | 65(45.5) | 39(27.3) |  |
| Marriage | Single | 4(6.9) | 14(24.1) | 29(50) | 11(19) | 0.005 |
|  | Married | 43(17.5) | 67(27.2) | 88(35.8) | 48(19.5) |  |
|  | others | 5(12.2) | 20(48.8) | 15(36.6) | 1(2.4) |  |
| Diabetes years | <1 | 4(12.1) | 14(42.4) | 12(36.4) | 3(9.1) | 0.034 |
|  | 1-5 | 9(7.2) | 39(31.2) | 52(41.6) | 25(20) |  |
|  | 6-10 | 9(9.5) | 29(30.5) | 46(48.4) | 11(11.6) |  |
|  | >11 | 33(19) | 57(32.8) | 56(32.2) | 28(16.1) |  |
| Family Diabetes | Yes | 29(9.6) | 100(33) | 121(39.9) | 53(17.5) | 0.023 |
|  | No | 23(20) | 37(32.2) | 42(36.5) | 13(11.3) |  |
| Ability in Diet plan |  | 7.27 | 7.52 | 8.49 | 8.21 | <0.001 |
| Ability in SMBG |  | | | | | |
| Knowing to use the devices | Yes | 42(11.5) | 109(29.9) | 151(41.4) | 63(17.3) | 0.002 |
|  | No | 10(16.7) | 30(50) | 17(28.3) | 3(5) |  |
| Knowing the device info | Yes | 45(11.5) | 121(31) | 159(40.8) | 65(16.7) | 0.007 |
|  | No | 6(18.2) | 18(54.5) | 8(24.2) | 1(3) |  |
| Knowing the level | Yes | 34(9.2) | 124(33.4) | 151(40.7) | 62(16.7) | <0.001 |
|  | No | 17(31.5) | 15(27.8) | 17(31.5) | 5(9.3) |  |
| DKQ |  | 11.13 | 15.02 | 16.21 | 17.66 | <0.001 |

SMBG: Self-Monitoring of Blood Glucose

DKQ: diabetes knowledge questionnaire

Chi-square test or ANOVA, P<0.05

Table S2 the variables with significant associations with different health literacy levels in acquiring health information dimension

|  |  | inadequate | Limited/ problematic | sufficient | excellent | p |
| --- | --- | --- | --- | --- | --- | --- |
|  |  | N(%)  mean | N(%)  mean | N(%)  mean | N(%)  mean |  |
| Age |  | 70.02 | 64.85 | 58.8 | 52.91 | <0.001 |
| Sex | Male | 47(18.4) | 20(7.8) | 126(49.2) | 63(24.6) | 0.038 |
|  | Female | 41(24.6) | 19(11.4) | 83(49.7) | 24(14.4) |  |
| Education | Primary | 40(64.5) | 11(17.7) | 11(17.7) | 0(0) | <0.001 |
|  | J high | 12(25) | 10(20.8) | 20(41.7) | 6(12.5) |  |
|  | S high | 32(18.3) | 12(6.9) | 99(56.6) | 32(18.3) |  |
|  | College | 5(3.5) | 6(4.2) | 82(57.3) | 50(35) |  |
| Marriage | Single | 6(10.3) | 6(10.3) | 27(46.6) | 19(32.8) | 0.013 |
|  | Married | 50(20.3) | 24(9.8) | 117(47.6) | 55(22.4) |  |
|  | others | 11(26.8) | 3(7.3) | 26(63.4) | 1(2.4) |  |
| Diabetes years | <1 | 6(18.2) | 2(6.1) | 22(66.7) | 3(9.1) | 0.003 |
|  | 1-5 | 16(12.8) | 13(10.4) | 62(49.6) | 34(27.2) |  |
|  | 6-10 | 17(17.9) | 4(4.2) | 55(57.9) | 19(20) |  |
|  | >11 | 50(28.7) | 20(11.5) | 72(41.4) | 32(18.4) |  |
| Ability in Exercise plan |  | 6.92 | 7.5 | 7.86 | 8.04 | 0.021 |
| Ability in Diet plan |  | 7.08 | 7.49 | 8.2 | 8.56 | <0.001 |
| Ability in SMBG |  | | | | | |
| Knowing to use the devices | Yes | 61(16.7) | 35(9.6) | 186(51) | 83(22.7) | <0.001 |
|  | No | 27(45) | 4(6.7) | 25(41.7) | 4(6.7) |  |
| Knowing the device info | Yes | 72(18.5) | 35(9) | 199(51) | 84(21.5) | 0.002 |
|  | No | 15(45.5) | 4(12.1) | 11(33.3) | 3(9.1) |  |
| Knowing the level | Yes | 64(17.3) | 34(9.2) | 191(51.5) | 82(22.1) | <0.001 |
|  | No | 23(42.6) | 5(9.3) | 20(37) | 6(11.1) |  |
| DKQ |  | 12.01 | 15.23 | 16.14 | 17.17 | <0.001 |

SMBG: Self-Monitoring of Blood Glucose

DKQ: diabetes knowledge questionnaire

Chi-square test or ANOVA, P<0.05

Table S3 the variables with significant associations with different health literacy levels in understanding health information dimension

|  |  | inadequate | Limited/ problematic | sufficient | excellent | p |
| --- | --- | --- | --- | --- | --- | --- |
|  |  | N(%)  mean | N(%)  mean | N(%)  mean | N(%)  mean |  |
| Age |  | 66.89 | 65.42 | 60.51 | 58.68 | 0.015 |
| Education | Primary | 6(9.7) | 6(9.7) | 39(62.9) | 11(17.7) | 0.002 |
|  | J high | 1(2.1) | 2(4.2) | 34(70.4) | 11(22.9) |  |
|  | S high | 9(5.1) | 3(1.7) | 116(66.3) | 47(26.9) |  |
|  | College | 3(2.1) | 7(4.9) | 75(52.4) | 58(40.6) |  |
| Marriage | Single | 2(3.4) | 3(5.2) | 39(67.2) | 14(24.1) | 0.02 |
|  | Married | 11(4.5) | 13(5.3) | 132(53.7) | 90(36.6) |  |
|  | others | 5(12.2) | 2(4.9) | 29(70.7) | 5(12.2) |  |
| Ability in Exercise plan |  | 8.06 | 5.94 | 7.63 | 7.94 | 0.026 |
| Ability in Diet plan |  | 8.19 | 6.89 | 7.83 | 8.41 | 0.022 |
| Ability in SMBG |  | | | | | |
| Knowing to use the devices | Yes | 13(3.6) | 14(3.8) | 221(60.5) | 117(32.1) | 0.025 |
|  | No | 4(6.7) | 5(8.3) | 42(70) | 9(15) |  |
| Knowing the device info | Yes | 14(3.6) | 15(3.8) | 239(61.3) | 122(31.3) | 0.026 |
|  | No | 2(6.1) | 4(12.1) | 23(69.7) | 4(12.1) |  |
| Knowing the level | Yes | 12(3.2) | 13(3.5) | 231(62.3) | 115(31) | 0.023 |
|  | No | 4(7.4) | 6(11.1) | 32(59.3) | 12(22.2) |  |
| DKQ |  | 10.63 | 13.53 | 15.12 | 16.98 | <0.001 |

SMBG: Self-Monitoring of Blood Glucose

DKQ: diabetes knowledge questionnaire

Chi-square test or ANOVA, P<0.05

Table S4 the variables with significant associations with different health literacy levels in assessing health information dimension

|  |  | inadequate | Limited/ problematic | sufficient | excellent | p |
| --- | --- | --- | --- | --- | --- | --- |
|  |  | N(%)  mean | N(%)  mean | N(%)  mean | N(%)  mean |  |
| Age |  | 66.01 | 58.84 | 57.67 | 54.2 | <0.001 |
| Education | Primary | 43(69.4) | 7(11.3) | 12(19.4) | 0(0) | <0.001 |
|  | J high | 19(39.6) | 4(8.3) | 24(50) | 1(2.1) |  |
|  | S high | 53(30.3) | 19(10.9) | 94(53.7) | 9(5.1) |  |
|  | College | 34(23.8) | 16(11.2) | 73(51) | 20(14) |  |
| Diabetes years | <1 | 14(42.4) | 4(12.1) | 13(39.4) | 2(6.1) | 0.001 |
|  | 1-5 | 29(23.2) | 20(16) | 62(49.6) | 14(11.2) |  |
|  | 6-10 | 28(29.5) | 5(5.3) | 57(60) | 5(5.3) |  |
|  | >11 | 79(45.4) | 16(9.2) | 70(40.2) | 9(5.2) |  |
| Ability in Exercise plan |  | 7.18 | 7.35 | 7.99 | 8.31 | 0.018 |
| Ability in Diet plan |  | 7.49 | 7.76 | 8.32 | 8.41 | 0.007 |
| Ability in treatments |  |  |  |  |  |  |
| Understand the drug plan | Yes | 132(33) | 45(11.2) | 193(48.2) | 30(7.5) | 0.045 |
|  | No | 12(60) | 0(0) | 8(40) | 0(0) |  |
| Ability in SMBG |  | | | | | |
| Knowing to use the devices | Yes | 113(31) | 42(11.5) | 182(49.9) | 28(7.7) | 0.002 |
|  | No | 34(56.7) | 4(6.7) | 20(33.3) | 2(3.3) |  |
| Knowing the device info | Yes | 128(32.8) | 42(10.8) | 190(48.7) | 30(7.7) | 0.041 |
|  | No | 18(54.5) | 4(12.1) | 11(33.3) | 0(0) |  |
| Knowing the level | Yes | 113(30.5) | 45(12.1) | 183(49.3) | 30(8.1) | <0.001 |
|  | No | 33(61.1) | 1(1.9) | 20(37) | 0(0) |  |
| DKQ |  | 13.5 | 16.28 | 16.34 | 17.17 | <0.001 |

SMBG: Self-Monitoring of Blood Glucose

DKQ: diabetes knowledge questionnaire

Chi-square test or ANOVA, P<0.05

Table S5 the variables with significant associations with different health literacy levels in applying health information dimension

|  |  | inadequate | Limited/ problematic | sufficient | excellent | p |
| --- | --- | --- | --- | --- | --- | --- |
|  |  | N(%)  mean | N(%)  mean | N(%)  mean | N(%)  mean |  |
| Age |  | 65.01 | 64.26 | 58.96 | 53.9 | <0.001 |
| Education | Primary | 31(50) | 10(16.1) | 21(33.9) | 0(0) | <0.001 |
|  | J high | 14(29.2) | 1(2.1) | 28(58.3) | 5(10.4) |  |
|  | S high | 36(20.6) | 19(10.9) | 107(61.1) | 13(7.4) |  |
|  | College | 28(19.6) | 6(4.2) | 87(60.8) | 22(15.4) |  |
| Marriage | Single | 11(19) | 2(3.4) | 35(60.3) | 10(17.2) | 0.008 |
|  | Married | 80(32.5) | 13(5.3) | 130(52.8) | 23(9.3) |  |
|  | others | 10(24.4) | 6(14.6) | 25(61) | 0(0) |  |
| Family Diabetes | Yes | 67(22.1) | 22(7.3) | 181(59.7) | 33(10.9) | 0.025 |
|  | No | 40(34.8) | 11(9.6) | 57(49.6) | 7(6.1) |  |
| Ability in treatments |  |  |  |  |  |  |
| Understand the drug plan | Yes | 97(24.2) | 29(7.2) | 234(58.5) | 40(10) | 0.001 |
|  | No | 7(35) | 6(30) | 7(35) | 0(0) |  |
| Ability in SMBG |  | | | | | |
| Knowing the level | Yes | 85(22.9) | 33(8.9) | 215(58) | 38(10.2) | 0.019 |
|  | No | 22(40.7) | 2(3.7) | 28(51.9) | 2(3.7) |  |
| DKQ |  | 13.57 | 15.25 | 15.95 | 17.25 | <0.001 |

SMBG: Self-Monitoring of Blood Glucose

DKQ: diabetes knowledge questionnaire

Chi-square test or ANOVA, P<0.05

Table S6 the variables with significant associations with different health literacy levels in communication dimension

|  |  | inadequate | Limited/ problematic | sufficient | excellent | p |
| --- | --- | --- | --- | --- | --- | --- |
|  |  | N(%)  mean | N(%)  mean | N(%)  mean | N(%)  mean |  |
| Age |  | 65.7 | 64.41 | 59.73 | 57.46 | <0.001 |
| Education | Primary | 16(25.8) | 9(14.5) | 32(51.6) | 5(8.1) | <0.001 |
|  | J high | 8(16.7) | 5(10.4) | 24(50) | 11(22.9) |  |
|  | S high | 24(13.7) | 12(6.9) | 107(61.1) | 32(18.3) |  |
|  | College | 6(4.2) | 17(11.9) | 75(52.4) | 45(31.5) |  |
| Marriage | Single | 7(12.1) | 3(5.2) | 36(62.1) | 12(20.7) | 0.032 |
|  | Married | 29(11.8) | 31(12.6) | 121(49.2) | 65(26.4) |  |
|  | others | 7(17.1) | 4(9.8) | 28(68.3) | 2(4.9) |  |
| Ability in Exercise plan |  | 6.61 | 8.05 | 7.6 | 8.24 | 0.004 |
| Ability in treatments |  |  |  |  |  |  |
| Understand the drug plan | Yes | 44(11) | 40(10) | 225(56.2) | 91(22.8) | 0.048 |
|  | No | 5(25) | 4(20) | 10(50) | 1(5) |  |
| DKQ |  | 12.91 | 15.07 | 15.26 | 17.38 | <0.001 |

DKQ: diabetes knowledge questionnaire

Chi-square test or ANOVA, P<0.05
